# Weekly induction intraperitoneal chemotherapy after primary surgical cytoreduction in patients with advanced epithelial ovarian cancer

**DOI:** 10.1186/1477-7819-4-4

**Published:** 2006-01-19

**Authors:** Rong Yu Zang, Zi Ting Li, Jie Tang, Xiao Huang, Shu Mo Cai

**Affiliations:** 1 Department of Gynecologic Oncology, Fudan University Cancer Hospital, Shanghai, China

## Abstract

**Background:**

Traditional intraperitoneal (IP) therapy administered simultaneously with intravenous (IV) chemotherapy in the primary setting has been well documented. This retrospective study was conducted to investigate the role of weekly IP therapy as an inducing intervention before front-line IV chemotherapy, particularly in patients with bulky residual disease after surgery.

**Methods:**

A total of 426 patients with advanced ovarian cancer treated between 1990 and 1999, were reviewed. Follow-up data were available in 409 patients. Of whom, 230 patients received postoperative weekly IP therapy with a median cycles of 4, other 179 patients who did not receive any IP therapy were used as the control group.

**Results:**

The median age of the patients was 51 years (range, 20–77 years). One hundred eighty-nine patients with stage III disease and 41 patients with stage IV disease were treated with postoperative IP therapy, respectively. Complications and toxicity were observed in 68 patients (29.5%), but there were no grade 4 toxicities and no patients died of complications or toxicities. In patients with residual disease > 1 cm, the median survival of those with IP delivery of chemotherapy and those without was 21.6 months and 18.8 months, respectively (hazard ratio [HR]= 0.69, *P *= 0.02). Whereas, in patients with residual disease ≤ 1 cm, the median survival was 46.8 months and 37.6 months, respectively (HR= 0.73, *P *= 0.09). Multivariate analysis suggested that the factors age ≤ 60 years, stage III, IP therapy and paclitaxel as front-line chemotherapy were associated with a better prognosis for patients with advanced ovarian cancer.

**Conclusion:**

Weekly postoperative IP therapy as an inducing intervention is practical for both physicians and patients with acceptable complications and associated with a lengthened survival of patients with advanced ovarian cancer. Whether this arm can be used in lieu of a traditional one needs further randomized trial to confirm the preliminary results.

## Background

Intraperitoneal (IP) therapy is the direct instillation of chemotherapy into the peritoneal cavity. First proposed by Dedrick *et al*, [1,2] IP arm is designed to maximize drug delivery to the tumor while avoiding many of the systemic toxicities associated with intravenous (IV) administration of the drug. It has been hypothesized previously that the high local concentration of cisplatin achievable within the peritoneal cavity after regional administration (10- to 20-fold greater than measured within the systemic compartment) would exert its maximum benefit in patients with microscopic residual disease or very small-volume macroscopic cancer at the time of IP drug delivery [3]. Support for this concept comes from early preclinical data where the depth of penetration of cytotoxic agents directly into the tumor or normal tissue after regional delivery has been measured in millimeter or less from the surface of the peritoneal lining [2,4-6]. A number of phase II trials of IP therapy have examined in the salvage setting in women with ovarian cancer have revealed that responses are almost exclusively observed in individuals with microscopic disease only or in those individuals whose maximal tumor diameter measures less than 0.5 to 1 cm [7,8]. Mature results are also available from two randomized phase III trials conducted by the Gynecologic Oncology Group (GOG) (GOG104, GOG 114) [9,10]. A landmark study, initiated in the mid 1980s and published in 1996, compared a regimen of IV cyclophosphamide plus IV cisplatin to a regimen of IV cyclophosphamide plus IP cisplatin [9]. In this trial, the median survival time of patients treated with IP cisplatin was improved from 41 to 49 months (*P *= 0.02) and the death HR was reduced by 24%, in favor of the regional treatment program. Although the authors claimed less toxicity observed in IP drugs delivery, 25% patients received IP therapy less than 4 cycles. In addition, a criticism of this trial is that 73% patients with residual disease ≤ 0.5 cm did not statistically benefit from IP therapy, in other words, just a cohort of 27% women with residual disease between 0.5–2 cm benefited most from this regional treatment program. In the second study (GOG 114), Markman *et al *[10], compared the new standard regimen of IV cisplatin plus IV paclitaxel to a regimen of moderately high-dose carboplatin followed by IP cisplatin plus IV paclitaxel. They documented the median survival time for women treated with IP therapy was improved from 52 to 63 months, and the death HR was reduced by 19%. One may argue a marginally significant survival improvement (*P *= 0.05) amounted to a remarkable 11-month prolongation. The results from that trial were challenged on the basis of the unknown impact of 2 cycles of carboplatin that was used before initiation of the IP therapy, concern about 18% of women received cycles of IP therapy. An editorial by McGuire [11] proclaimed that, in the second trial, savage therapies that might influence survival end points were not controlled, and how frequently salvage therapies administered before disease progression that might also influence progression-free survival was not divulged by the authors.

These results, however, do not lead to the widespread practice of IP therapy. Why? Controversies remain both over safety and efficacy of this approach. Consequently, there is likely considerable room for improvement with respect to study design. Traditional IP therapy was delivered simultaneously with IV chemotherapy and repeated every three weeks. With much different to that in the literature, this series was carried out to evaluate the role of weekly IP therapy delivered independently with IV chemotherapy.

## Patients and methods

We retrospectively reviewed the records of 426 patients who underwent primary surgery for advanced epithelial ovarian cancer between 1990 and 1999 at Fudan University Cancer Hospital. Patients received postoperative IP therapy were compared with those without any IP therapy. IP therapy repeated every week was followed by systemic IV chemotherapy. Follow-up data were available for 409 patients who formed the basis of this study.

Regular IP therapy used independently with IV chemotherapy began from 2–7 days after surgery. Two hundred thirty patients (56.2%) received 2–9 courses (median, 4 courses) of postoperative IP therapy. One hundred and five women (25.7%) received 1–3 courses (mean, 3 courses) of neoadjuvant IP therapy, which was delivered before primary surgery and not considered as an inducing intervention. IP therapies delivered after systemic front-line intravenous chemotherapy such as those for consolidation or salvage intent were not included in this study. IP arm consisted of platinum-based combination therapy administered through a Tenckhoff, or a Port A, or Groshong catheter. Cisplatin, 60 mg/m^2^, and 5-FU, 800–1000 mg/m^2^, and/or mitomycin C 6 mg/m^2^, or etoposide, 100 mg/m^2^, were given in 1.5–2 liters of normal saline and administered IP as fast as possible, usually within 2 hrs. Hydration was given with 1000–1500 ml 5% glucose in 4 hrs.

The cut-off of residual disease in the greatest dimension after primary surgery was 1 cm. Statistical analysis included tests for associations between potential prognostic factors and between prognostic factors and survival. Survival probabilities were estimated by Kaplan-Meier methods, and prognostic factors for survival were evaluated by a log-rank test (univariate) or a Cox proportional hazard model (multivariate).

## Results

### Patient characteristics

The median age of the patients was 51 years (range, 20–77 years). The baseline patient characteristics of the patients are listed in Table 1. Of the 409 eligible patients, 331 and 78 patients had International Federation of Gynecology and Obstetrics (FIGO) stage III and stage IV disease, respectively. Distribution of perioperative IP therapy by stages was as follows, neoadjuvant IP therapy: stage III, 71; IV, 34; and postoperative IP therapy: stage III, 189; IV, 41. One hundred ninety-one women had residual disease of ≤ 1 cm after primary surgery, and 218 patients had residual disease > 1 cm.

**Table 1:** Baseline Patient Characteristics

Characteristics	IP therapy	Without IP therapy
	(*n *= 230)	(*n *= 179)
Age		
<41	33	27
41–60	172	138
>60	14	25
FIGO stage		
IIIa	19	8
IIIb	22	6
IIIc	148	128
IV	41	37
Residual disease		
≤ 1 cm	123	68
> 1 cm	107	111
Grade		
1	8	4
2	100	67
3	122	107
Not available	0	1
Histology		
Serous	116	88
Mucinous	13	18
Endometrioid	25	16
Unspecified adenocarcinoma	44	37
Mixed	7	7
Clear cell	18	7
Others	7	6

Abbreviation: IP, intraperitoneal; FIGO, International Federation of Gynecology and Obstetrics.

### Complications and toxicity

Table 2 outlines the major complications and toxicities encountered by IP chemotherapy after surgery. Complications and toxicity were observed in 68 patients (29.5%). There were no grade 4 toxicities and no patients died of complications or toxicities.

**Table 2:** Complications and toxicities associated with IP therapy*

**Complications and toxicity**	**no.**	**%**
Catheter related		
Catheter malfunction	21	9.1
Catheter-induced infection	5	2.2
Toxicities (Grade 3)		
Neuropathy	1	0.4
WBC	7	3.0
Platelets	2	0.9
Other hematologic	13	5.7
Creatine clearance	1	0.4
Gastrointestinal	10	4.3
Others		
Chemical peritonitis	2	0.9
Severe abdominal pain	6	2.6
Total	68	29.5

Abbreviation: IP, intraperitoneal; WBC: white blood cell.

* IP used as neoadjuvant therapy is not included.

### Survival

**Figure 1:**
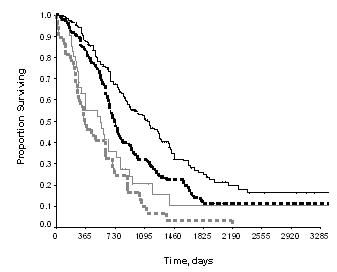
Overall survival by IP therapy. Black lines: stage III ovarian cancer, IP therapy (the solid line) vs. without IP therapy (the dash line), χ^2 ^= 9.41, *P *< 0.01; Gray lines: stage IV ovarian cancer, IP therapy (the solid line) vs. without IP therapy (the dash line), *P *> 0.05

**Figure 2:**
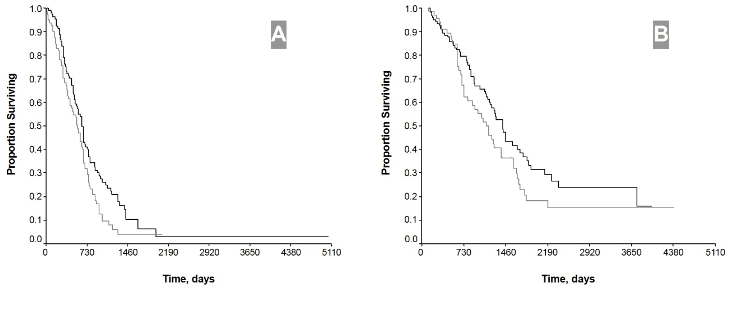
Overall survival by IP therapy and residual disease after primary surgery. IP therapy vs. without IP therapy in patients with residual disease > 1 cm (HR = 0.69, *P *= 0.02) (**A**) and residual disease ≤ 1 cm (HR = 0.73, *P *= 0.09) (**B**), respectively

The median follow-up was 37 months (range, 3–169 months). Neoadjuvant IP therapy was not associated with the prolongation of survival in patients with advanced ovarian cancer (*P *> 0.05). When stratified by FIGO stage, patients with stage III disease treated with postoperative IP therapy (χ^2 ^= 9.41, *P *< 0.01) rather than those with stage IV disease (*P *> 0.05) benefited from postoperative IP therapy (Figure 1). When patients stratified by the size of residual disease, in suboptimal group (residual disease >1 cm), the median survival of those with IP arm vs. those without was 21.6 months vs.18.8 months, respectively (hazard ratio [HR] = 0.69, *P *= 0.02). (Figure 2A). In optimal group (residual disease ≤ 1 cm), no statistical differences were seen with respect to survival if patients were sub-grouped in terms of IP therapy or not (median 46.8 months versus 37.6 months, respectively; HR = 0.73, *P *= 0.09). (Figure 2B).

Table 3 summarizes the results of multivariate analysis, which suggested that, when it was stratified by residual disease (residual disease ≤ 1 cm and >1 cm), the factors age ≤ 60 years, stage III, IP therapy, and paclitaxel as front-line chemotherapy were associated with a better prognosis for patients with advanced ovarian cancer. However, neoadjuvant IP therapy was not an independent survival determinant.

**Table 3:** Multivariant analysis of effects on overall survival stratified by residual disease after primary surgery.

**Variable**	**Harzard**	**95%CI**	***P *value**
Age >60	1.26	1.02–1.57	0.03
FIGO stage IV	4.84	2.73–8.57	<0.01
Initial paclitaxel*	0.80	0.71–0.90	<0.01
IP chemotherapy	0.71	0.56–0.90	<0.01

Abbreviation: IP, intraperitoneal; 95%CI, 95% confidential interval; FIGO, International Federation of Gynecology and Obstetrics.

* Paclitaxel as front-line chemotherapy.

## Discussion

### The rationale of independent weekly IP chemotherapy

In the past two decades, we used IP therapy between primary surgery and front-line IV chemotherapy, which was repeated every week with a median 4 cycles in total. Such an approach held significant promise in both patients and the physicians in our hospital. One may argue the rationale of independent weekly IP intervention. It was based on the observations that the probability of intestinal adhesion increased with the time going after primary surgery and IP therapy itself might cause the adhesion. During secondary surgery we also observed that adhesion occurred more frequently in patients with IP therapy than those with IV therapy alone. Profound pharmacokinetic results have documented that higher intracellular drug levels result from direct tumor penetration and uptake. Once the adhesion occurring, it is difficulty for active drugs to pass the surrounded tissues into tumor nodes. Abdominal pain was common seen in patients with adhesion. That was one of the reasons why some patients were unwilling to receive IP therapy. We feel that weekly IP therapy is more practical than the traditional one, which is used simultaneously with intravenous chemotherapy. So we usually use this arm within 4 weeks after primary surgery. To our knowledge, it is the first report to advocate IP therapy used weekly, independently with IV therapy as a chemotherapeutic inducing intervention for advanced ovarian cancer.

### Survival impacts of weekly IP chemotherapy

To our surprise, survival benefit from weekly IP therapy is observed particularly in patients with suboptimal surgical cytoreduction, and this review documents an 2.8 months improvement in survival (*P *= 0.02) and a 31% reduction in the risk of death in patients treated with induction IP arm compared to those with upfront IV arm. In patients with optimal surgical cytoreduction an 9.2 months improvement in survival is observed, but it does not reach a statistical significantly difference (HR = 0.73, *P *= 0.09).

It is different from that reported in the literature, but the results are quite exciting. The possible explanations for that might be as follows: (1) weekly IP delivery of chemotherapy for about 3 cycles plays different roles of chemotherapeutic induction and enhance the efficiency of the following IV chemotherapy in patients with suboptimal and optimal surgical cytoreduction. In patients with optimal cytoreduction, IP chemotherapy can eradicate the small lesions, particularly those of <0.5 cm. The question is how IP arm works in patients with bulky residual disease. Till now, in western countries, most oncologists just advocate IP chemotherapy used in micro-residual disease. Whereas, widespread microscopic residual disease remain a problem as well as the bulky lesions when suboptimal cytoreduction happens. It is indefinite whether or not IP arm has an effect on bulky nodes, but drugs in peritoneal can eradicate microscopic disease for those suboptimal surgical results. At this point, weekly IP chemotherapy plays an inducing role when it is followed by IV chemotherapy. (2) Figure 2A shows some patients with suboptimal cytoreduction treated with IP arm survived more than 14 years, whereas in optimal cytoreduction group no patients treated with IP chemotherapy survived more than 12 years, and that is why the two curves in the former are significantly separated. Although this series is a retrospective observation, noteworthy is the significant long-term survival benefit from IP delivery of chemotherapy. The curves of both Figure 2A and 2B tend to separate more significantly in those after 2 years follow-up than those within 2 years. In addition, when categorized by the factor residual disease, multivariate analysis suggests that IP therapy remains one of the survival determinants. It may be translated that 4 cycles IP therapy delivered within 5 weeks after surgery is mainly associated with long-term survival in patients with advanced ovarian cancer. Our previous studies have documented that 6 cycles IV chemotherapy delivered in 18 weeks just improve the 3-year survival [12,13]. Consequently, we are quite encouraged by the present results because how to improve long-term survival remains the major problem in the treatment of patients with advanced ovarian cancer. Heated/ hyperthermia intraperitoneal chemotherapy (HIPEC), which was first established by Spratt *et al*, in the late 1970s, had recently been documented to have a survival advantage and improvement of quality of life for patients with pseudomyxoma peritonei, colorectal-, gastric and ovarian cancer [14-16]. However, HIPEC will not used as a regular arm for patients with ovarian cancer because of its inconvenient.

## Conclusion

The present results suggest that weekly IP delivery of chemotherapy for 4 cycles should be a standard care for ovarian cancer. Well-designed randomized controlled trials should be conducted to confirm the results.

## Competing interests

The author(s) declare that they have no competing interests.

## Authors' contributions

**RYZ **collected the data, patients' follow-up and drafted the article.

**ZTL, JT, XH, SMC **collectively designed the study, participated in collection of data and revising the article.

## Funding Source

This work supported in part by the New Star Project of the Shanghai Health Bureau (R.Y.Z.).
